# Importance of Zinc Transporter 8 Autoantibody in the Diagnosis of Type 1 Diabetes in Latin Americans

**DOI:** 10.1038/s41598-017-00307-4

**Published:** 2017-03-16

**Authors:** Karla Fabiana Brasil Gomes, Cintia Semzezem, Rodolfo Batista, Rosa Tsuneshiro Fukui, Aritania Sousa Santos, Márcia Regina Correia, Maria Rita Passos-Bueno, Maria Elizabeth Rossi da Silva

**Affiliations:** 1 Laboratório de Carboidratos e Radioimunoensaio –LIM 18, Hospital das Clínicas da Faculdade de Medicina da Universidade de São Paulo. Av Dr. Arnaldo 455, 01246-903 São Paulo, Brazil; 2 Departamento de Genética e Biologia Evolutiva, Instituto de Biociências da Universidade de São Paulo. Rua do Matão, 277, 05422-970 São Paulo, São Paulo Brazil

## Abstract

There is a scarcity of data of zinc transporter-8 autoantibody (ZnT8A) on mixed populations such as Brazilian. Therefore, we evaluated the relevance of ZnT8A for type 1 diabetes (T1D) diagnosis and the role of ZnT8 coding gene (SLC30A8) in T1D predisposition. **P**atients with T1D (n = 629; diabetes duration = 11 (6–16) years) and 651 controls were genotyped for SLC30A8 rs16889462 and rs2466295 variants (BeadXpress platform). ZnT8 triple antibody was measured by ELISA; glutamic acid decarboxylase (GAD65A) and protein tyrosine phosphatase (IA-2A) autoantibodies by radioimmunoassay. Results: Znt8A was detected in 68.7% of recent-onset T1D patients and 48.9% of the entire patient cohort, similar to GAD65A (68.3% and 47.2%) and IA-2A (64.8% and 42.4%) positivities respectively. ZnT8A was the only antibody in 8.4% of patients. Znt8A and IA2A frequencies and titers were independent of gender and ethnicity, whereas GAD65A titers were greater in females. The diabetes duration-dependent decline in ZnT8A frequency was similar to GAD65A and IA-2A. The SLC30A8 rs2466293 AG + GG genotypes were associated with T1D risk in non-European descents (56.2% × 42.9%; p = 0.018), and the GG genotype with higher ZnT8A titers in recent-onset T1D: 834.5 IU/mL (711.3–2190.0) × 281 IU/mL (10.7–726.8); p = 0.027. Conclusion ZnT8A detection increases T1D diagnosis rate even in mixed populations. SLC30A8 rs2466293 was associated with T1D predisposition in non-European descents.

## Introduction

The major molecular targets of type 1 diabetes (T1D) autoimmunity have been identified by islet autoantibody research in humans, providing insight into the etiology, pathogenesis, and natural history of the disease. Humoral auto-reactivity typically precedes clinical disease by months or years, and the progression to clinical manifestations is marked by intramolecular and intermolecular epitope spreading^1, 2^. Beyond diagnosis, autoantibody determinations may also be useful for assessing the immunological impact of therapeutic interventions targeting autoreactive T- and B-cells^3^.

Zinc transporter-8 (ZnT8), the product of the *SLC30A8* gene, is a secretory granule membrane protein of the pancreatic beta cells that was recently identified as an autoantigen in T1D^4^.

Autoantibodies to ZnT8 (ZnT8A) complement the established set of type 1 diabetes-associated autoantibodies to insulin (IAA), glutamic acid decarboxylase (GAD65A), and protein tyrosine phosphatase (insulinoma-2 antigen antibody, IA-2A), which are important for predicting and confirming the T1D diagnosis^5^. ZnT8A has been detected in 60–80% of Caucasian^4–9^ and 24–70% of Asian populations with T1D^10, 11^. As several patients with presumed diagnosis of T1D lack GAD65A and IA2A autoantibodies, establishing the autoimmune basis sometimes becomes difficult^1^. Inclusion of ZnT8A determination reduces the proportion of patients with negative autoantibodies and increases the diagnostic sensitivity to over 90% for new onset cases of T1D in Caucasians^7, 9^.

It is known that genetic and environmental factors contribute to the different incidence rates and clinical characteristics of T1D among populations. The Brazilian population is one of the most heterogeneous in the world with a wide degree of admixture, with major contributions coming from European ancestry (0.771), followed by African (0.143) and Amerindian ancestries (0.085)^12^.

As data on autoimmune manifestations in the Brazilian population are scarce, the aim of our study was to estimate the prevalence of ZnT8A in T1D patients and determine the utility of ZnT8A as a T1D immunity marker, either alone in GAD65A- and IA-2A-negative subjects or in conjunction with them. The humoral response to ZnT8 was also evaluated considering the age at diabetes onset, disease duration, gender, ethnicity and *SLC30A8* variants. Variation in *SLC30A8* may affect zinc accumulation in insulin granules and the stability, storage, and secretion of insulin^13^.

Amino acid changes at position 325 of ZnT8 coding gene *SLC30A8*, which are represented by arginine (ZnT8-R), tryptophan (ZnT8-W) and glutamine (ZnT8-Q), are determined by the rs13266634 and rs16889462 missense mutations^6, 7^, which represent three antigenic epitopes. Both SNPs are in linkage disequilibrium^14^ and together with rs2466295 seem to be related to the T2D risk and response to therapy^14–16^.

However, the role of *SLC30A8* in T1D predisposition is still controversial. Genome-wide association studies did not confirm the association between *SLC30A8* and T1D^17^. The CC genotype in the most studied variant of SLC30A8, rs13266634, was mainly related to a younger age at T1D diagnosis in Germany^18^ and non-Swedish immigrants^19^, whereas the same C allele determined higher stimulated C-peptide levels during the first year following T1D diagnosis^20^. We therefore evaluated the roles of *SLC30A8* rs16889462 and rs2466295 in T1D pathogenesis.

## Results

The demographic and laboratory data of the patients with T1D and of the health controls are provided in Table 1. The age at diagnosis of T1D patients (median and interquartile range-25^th^ and 75^th^) was 11 (6–16) years, and the diabetes duration was 11 (3–19 years) years.

**Table 1:** Demographic and laboratory characteristics of patients with T1D and controls.

	T1D	Controls	OR (CI)	*P*
	N = 629	N = 651		
Age (years)	23 (14–33)	25 (20–35)		<0.0001
Age at diagnosis (years)	11 (6–16)			
Years of disease	11 (3–19)			
Female (%)	58.7	37.6	1.99 (1.48–2.67)	<0.0001
European Ancestry (%)	80.1	60.7	1.96 (1.42–2.72)	<0.0001
Glycemia (mg/dL)	163.0 (96–261)	84.0 (77–90)		<0.0001
HbA1c (%)	8.0 (7.0–9.6)	5.4 (5.1–5.6)		<0.0001
ZnT8Ab titer IU/mL)	14.0 (2.0–244.0)	1.0 (0.0–3.0)		<0.0001
ZnT8A frequency (%)	48.3	1.9	55.6 (29.7–104.1)	<0.0001
GAD65A titer IU/mL)	0.9 (0.2–7.2)	0.0 (0.0–0.0)		<0.0001
GAD65A frequency (%)	47.2	1.7	53.4 (28.9–98.6)	<0.0001
IA-2A titer IU/mL)	0.3 (0.1–2.9)	0.0 (0.0–0.1)		<0.0001
IA-2A frequency (%)	42.4	1.6	42.3 (22.9–78.4)	<0.0001

Data are presented as median and (inter-quartile range). HbA1c = glycated hemoglobin; ZnT8A = Zinc transporter-8 autoantibody; GAD65A = glutamic acid decarboxylase 65 autoantibody, IA-2A = tyrosine phosphatase autoantibody.

As expected, HbA1c levels, glucose levels, islet autoantibody titers and frequencies were higher in the patients (p < 0.001). European ancestry and females prevailed among the T1D patients (p < 0.001).

ZnT8A was the only autoantibody observed in six control subjects (1.9%, 3F:3M) aged 1.9 to 27 years, who tested negative for GAD65A and IA-2A, and its frequency did not differ from that of GAD65A (1.7%) or IA2-A (1.7%) in the controls (p > 0.05). The normal value of ZnT8A in our cohort (n = 321) was defined as ≤16 u/mL, considering 3 SD.

Znt8A was detected in 48.9% of all patients, with median levels of 14 IU/mL (2–244). Znt8A positivity was similar to that of GAD65A (47.2%) and IA-2A (42.4%).

ZnT8A titers were similar between females and males: 12 (1–235) × 17 (2–290) IU/mL; p = 0.116 and between those of European and non-European ancestry: 12 (2–206) × 22 (2–380) IU/mL; p = 0.235. Titers of IA-2A also did not differ between females and males: 0.3 (0.12–2.87) × 0.4 (0.2–2.92) IU/mL; p = 0.384, and between whites and non-whites: 0.33 (1–2.5) × 0.4 (0.2–3.5); p = 0.168, whereas GAD65A titers were greater in females: 1 (0.25–11.28) × 0.7 (0.25–3.57) IU/mL; p = 0.022, but did not differ between those from European and non-European ancestry 0.8 (0.25–7.49) × 1.01 (0.25–6.58) iIU/mL; p = 0.779.

The frequencies of the three antibodies were independent of gender and ethnicity.

ZnT8A was similarly associated with GAD65A (30.2% of patients) and IA-2A (33.8%). Patients tested positive for ZnT8A plus IA-2A (33.8%) more frequently than for GAD65A plus IA-2A (26.7%) (OR = 1.403 (CI:1.073–1.835); Pc = 0.0396). IA-2A (but not GAD65A) positivity predicted positivity for ZnT8A (OR = 5.79; CI = 3.03-11-08; p < 0.001). There was also a positive correlation of ZnT8A levels with GADA (r = 0.307; p < 0.001) and IA2-A levels (r = 0.553, p < 0.001).

### Islet Autoantibody profile over time

**Figure 1:**
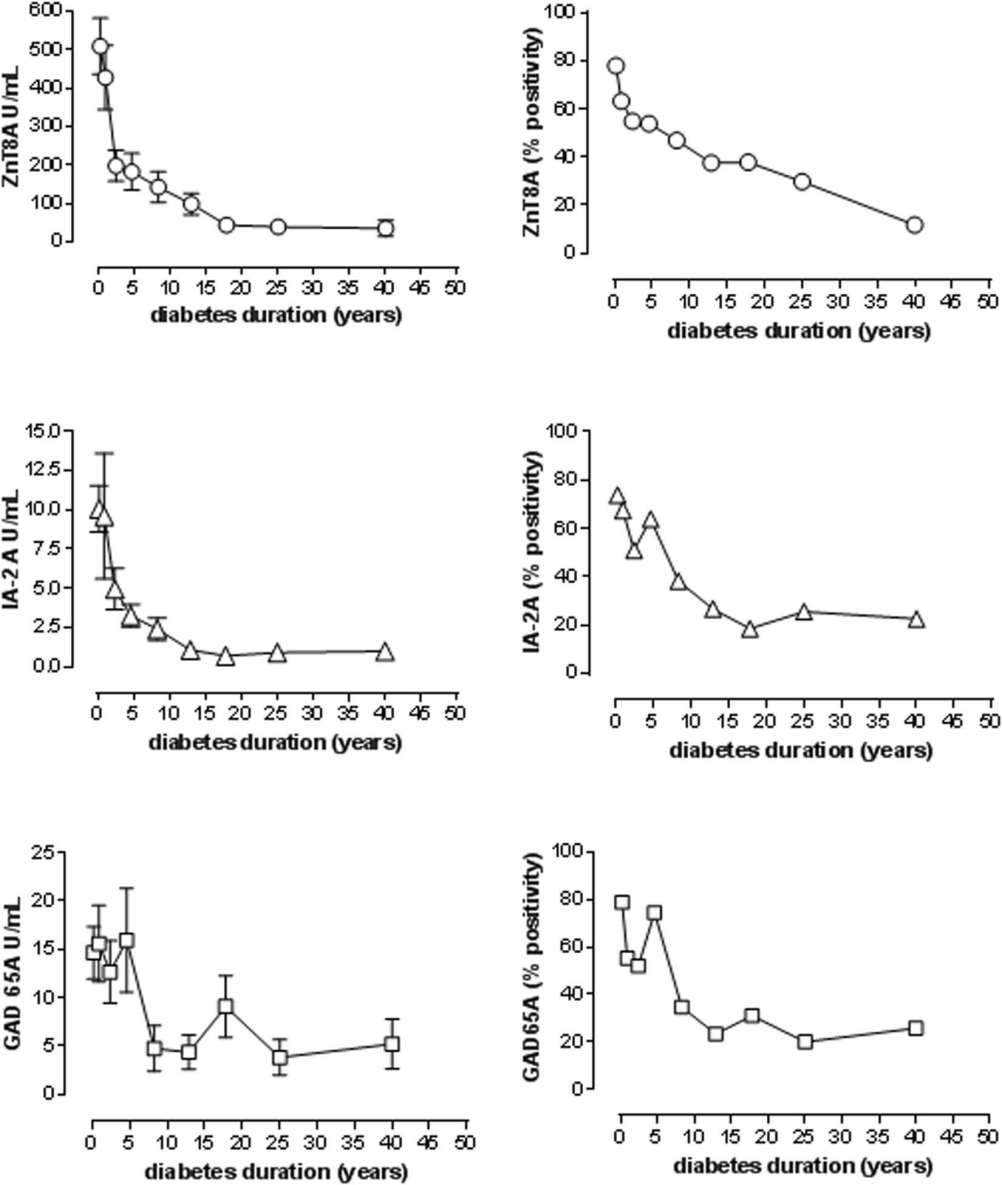
Mean levels (U/mL) and frequencies of autoantibodies against zinc transporter-8 (ZnT8A), glutamic acid decarboxylase (GAD65A) and tyrosine phosphatase (IA-2A) according to diabetes duration (years).

The frequency of the three islet autoantibodies was higher in recent-onset T1D patients and similar between them: ZnT8A (68.7%) × GAD65A (68.3%) × IA-2A (64.8%). The prevalence of all three autoantibodies declined in a similar way in the years following diagnosis and was more intense after the first year (Fig. 1). A duration-dependent decline in autoantibody titers was also observed for ZnT8A (rho = −0.44), IA-2A (rho = −0.33) and GAD65A (rho = −0.32); p < 0.001.

ZnT8A titers correlated negatively with the age at blood collection (r = −0.369; p < 0.0001) and diabetes duration (r = −0.439; p < 0.0001) but not with the age at diagnosis (r = −0.006; p = 0.91). When considering only those patients with recent diabetes onset (within less than two years of diagnosis), ZnT8A titers were negatively correlated with diabetes duration (r = −0, 273; p = 0.002) but again, not with the age at diagnosis (r = 0.017; p = 0.85).

### Importance of islet autoantibodies for the diagnosis of autoimmune diabetes

ZnT8A was detected in 26.1% of T1D patients (aged 11.4 ± 6.9 years) who tested negative for other islet autoantibodies. The inclusion of ZnT8A increased the detection rate of beta-cell autoimmunity and T1D from 67.6% to 76% in the entire cohort.

Among patients who were positive for only one islet autoantibody, ZnT8A, GAD65A and IA-2A were found in 8.4%, 12.5% and 5.9%, respectively. Thus, the determination of ZnT8A allowed the additional diagnosis of T1D in 8.4% of patients. Only 7.8% of recent-onset T1D patients (within 2 years’ duration) remained autoantibody-free.

### Influence of SLC30A8 susceptibility on islet autoantibody frequency

The SLC30A8 rs2466293 and rs16889462 genotypic frequencies were consistent with the Hardy-Weinberg distribution and were independent of gender but not of ethnicity. Individuals of non-European descent had a lower prevalence of rs2466295 AG + GG genotypes (47.5 × 56.3%; OR = 0.423, CI = 1.108–1.828; p = 0.011) but a greater prevalence of rs16889462 AG (6.3 × 2.8%; OR = 2.329, CI = 1.295–4.189; p = 0.0073) when compared with individuals of European descent.

Further, in non-whites, the rs2466293 AG + GG genotypes were associated with risk of T1D, prevailing in patients (56.2%) when compared with controls (42.9%); p = 0.018; OR = 1.711; CI = 1.095–2.672. The GG genotype also conferred greater ZnT8A titers: 834.5 (711.3–2190) × 281 IU/mL (10.75–726.8); p = 0.027 in recent-onset T1D (<2 years of duration).

The rs16889462 variant did not influence T1D susceptibility, ZnT8 titer or frequency after regression analysis. None of the SLC30A8 variants affected the prevalence or titers of GAD65A or IA-2A or the age at onset of T1D. The median age at disease onset was between 10 and 11 years for each SLC30A8 genotype, and there was no trend for any association in the youngest group (lower than 6 years).

## Discussion

The prevalence of ZnT8A in comparison with other pancreatic autoantibodies and the phenotypic characteristics of T1D patients positive for ZnT8A were determined, as well as the utility of ZnT8A as a marker of autoimmunity, either alone in antibody-negative subjects or in conjunction with GAD65A and IA2A.

Considering that the majority of studies in this field included Caucasians^4–9^ or Asians^10, 11^, the immunogenetic profile of admixtured populations such as ours remains unclear. Therefore, this analysis can add knowledge on T1D manifestations.

European ancestry prevailed in our population. The median age at T1D diagnosis was similar to that reported for Caucasians, but the distribution of gender differed. The highest prevalence of females had already been noticed by our group^21^ and by Braga *et al*.^22^ in the Brazilian type 1 diabetes Study Group, which contrasts with series that did not report differences between genders or that reported an even greater frequency in men with T1D^1^.

As expected, the prevalence of ZnT8A (68.7%) was higher in recently diagnosed T1D (within 2 years of diagnosis) and had the same sensitivity as GAD65A (68.3%) and IA-2A (64.8%). These data are consistent with the prevalence of ZnT8A reported in Caucasians^5, 9, 23, 24^, but greater than in those of Asian ancestry (26%)^25^.

ZnT8A was also confirmed as a biomarker of T1D similar to GAD65A and IA-2A, even after many years had elapsed since diagnosis - the median diabetes duration was 11 (3–19) years. The more frequent association of ZnT8A plus IA-2A positivity in comparison with that of ZnT8 plus GAD65A, which was previously observed in new onset T1D patients^25^, was maintained in our study, despite the long diabetes duration.

The frequency and titers of ZnT8A and IA-2A were unrelated to gender and ethnicity, differing from GAD65A titers which were higher in females.

The duration-time decline of the frequency of the three autoantibodies was very similar in the 30 years of follow up. After this time, approximately 20% of the patients persisted as positive for at least 1 autoantibody. There are few reports comparing the persistence of the three islet autoantibodies in such a long follow up. Wenzlau *et al*.^26^ found similar data for GAD65A and IA-2A positivity, but the data were lower for Znt8A (6.7%).

In contrast, autoantibody titers followed up within 2 years of clinical diabetes onset suggested a faster decline for ZnT8A titers (72.4%) than for IA-2A (53.4%) and GAD65A (19.5%), as previously reported^24^.

ZnT8A titers correlated negatively with the age at blood collection (r = −0.369; p < 0.0001) and diabetes duration (r = −0.439; p < 0.0001) but not with the age at diagnosis (r = −0.006; p = 0.91), even after considering only those patients with a recent diabetes onset.

This discrepancy might be due to the age-dependent bimodal distribution of Znt8A, which is less common among individuals aged 0–5 years^23^ and those over 20 years of age at blood draw compared with those aged 6–20 years^7, 9–11^.

ZnT8A was detected in 26.1% of T1D patients who tested negative for the two other islet autoantibodies. The inclusion of ZnT8A in the panel of diabetes-associated autoantibodies increased the sensitivity for beta-cell autoimmunity from 67% to 76% in the entire cohort. Among patients positive for only one islet autoantibody, ZnT8A, GAD65A and IA-2A were the only detectable markers of autoimmunity in 8.4%%, 12.5% and 5.9%, respectively. Thus, the determination of ZnT8A allowed the additional diagnosis of DM1 in 8.4% of the patients. In recent-onset T1D patients, only 7.8% remained negative for the three autoantibodies. Overall, the combined measurement of GAD, IA-2 and ZnT8 autoantibodies resulted in a diagnostic sensitivity for autoimmune diabetes of 92.2% at T1D onset. These results are in agreement with previous reports^7, 9^ or even slightly higher^23, 25, 27^ particularly for Asians^25^, and they confirm that the measurement of ZnT8 autoantibodies is important for the diagnosis of recent or long duration T1D patients, even in a multiethnic population

Variants of the ZnT8 coding gene, *SLC30A8*, which predisposes to T1D, have already been searched with contradictory results^18, 10^, whereas the rs16889462 and rs2466293 variants have been mainly related to impaired glucose metabolism and T2D risk in Europeans and Asians^14–16, 28^.

The SNPs rs2466293 (a 3′UTR variant) and rs16889462 (a missense variant determining a single amino acid variation at position 325 of the cytosolic segment of ZnT8, which codes for glutamine (CAG)^6, 7^ were not in linkage disequilibrium. Their distribution was independent of gender but not of ethnicity. Individuals of non-European descent had a lower prevalence of rs2466293 AG + GG genotypes (OR = 0.423, p = 0.011) but a greater prevalence of rs16889462 AG (OR = 2.329, p = 0.0073) when compared with individuals of European descent, as expected. The rs2466293 G allele is less common in Africans than Europeans (12% × 37%), whereas the rs16889462 A allele is nearly absent in Europeans (www.ensembl.org). This ancestry-related difference is thought to be reflected in our data.

In non-whites, the rs2466293 AG + GG genotypes were associated with T1D risk, prevailing in the patients (56.2%) when compared with the controls (42.9%, p = 0.018; OR = 1.711). The GG genotype also conferred greater ZnT8A titers: 834.5 (711.3–2190) × 281 IU/mL (10.75–726.8); p = 0.027 in recent-onset T1D (<2 years of duration). A similar trend for ZbT8A titers (not significant) was observed when analyzing the entire cohort. The decrease in beta-cell function due to the rs2466293 minor allele^28^ associated with the autoimmune insult could have precipitated the diabetes manifestation in this group. Another possibility is that the SNP modulates miRNA-directed regulation of gene expression^29^.

A trend toward higher frequency of anti-ZnT8A conferred by the rs16889462 AG genotype (75%) when compared with GG (46.7%) in patients with T1D was not confirmed after including gender, ancestry and diabetes duration as covariates in the analysis (p = 0.078; OR = 2.973; CI: 0.884–9.997), perhaps due to the low prevalence of the AG genotype (4.7% of patients).

Different ancestries could explain why a relationship of the variants with T1D was absent in Caucasians. These data suggest that genetic susceptibility to beta-cell dysfunction in the presence of autoimmunity may favor the progression of the disease. Linkage disequilibrium with rs13266634, linked to the specificity of ZnT8A^5, 7^ or with other variants related to beta-cell function, are probably implicated in our results.

None of the SLC30A8 variants influenced the prevalence of GAD65A or IA-2A or the age at onset of T1D. No genotype was associated with an early manifestation of diabetes.

Our study has limitations. Its cross-sectional nature implies that not all patients underwent autoantibody determinations at diagnosis or in the follow up. However, it is still informative considering that few studies have simultaneously compared the frequency of the three autoantibodies in such a long follow up (30 years). The groups were not homogeneous regarding age, gender, ancestry and BMI and these differences may have had an impact on the results.

## Conclusion

The ZnT8A triple autoantibody assay increased the detection rate of beta-cell autoimmunity and T1D from 67.6% to 76% in the entire cohort and was the only islet autoantibody in 8.4% of the patients. The frequency of Znt8A was similar to that of GAD65A and IA-2A and was independent of gender or ethnicity. Therefore, measurements of ZnT8 autoantibodies are important for Type 1 diabetes diagnosis and can be considered a marker of T1D autoimmunity in mixed populations such as ours. SLC30A8 rs2466293 seems to predispose to T1D risk in individuals of non-European descent.

## Materials and Methods

### Subjects

Patients with T1D (n = 629), which was defined according to American Diabetes Association criteria^30^, and healthy controls (n = 651) were analyzed. T1D diagnosis was based on the clinical symptoms of diabetes (weight loss, polyuria, polydipsia) or ketoacidosis at diagnosis; the need for permanent insulin therapy since diagnosis; and the presence of one or more islet autoantibodies. Autoantibody determinations started a few years ago. For this reason, some patients underwent this dosage many years after the diagnosis. The vast majority of patients were treated at the Clinical Hospital, and a small number of patients were referred by endocrinologists participating in the study. The medical records of patients always included the age at diagnosis and clinical features. Those who had any suspicion of non-autoimmune diabetes were not included in the cohort.

Healthy controls were also recruited in São Paulo through referrals from blood bank donors, hospital staff, the army and through announcements in the media. Healthy controls had normal blood glucose and glycosylated hemoglobin levels and no family history of diabetes or any other autoimmune disease.

Both groups were genotyped for *SLC30A8* variants (Table 1). Blood glucose, glycosylated hemoglobin and C-peptide levels were determined in both patients and controls. ZnT8 autoantibodies were determined in 414 patients with T1D and 320 healthy controls and were compared with those of GAD65A and IA-2A of 699 patients with T1D and 786 healthy controls. The approval of the Ethical Committee of the Hospital das Clínicas da Faculdade de Medicina da Universidade de São Paulo was obtained before informed consent was signed from participants and/or parents.

The study was approved by Ethical Committee of Faculdade de Medicina da Universidade de São Paulo and all methods were performed in accordance with the relevant ethical guidelines and regulations.

### Glucose, glycosylated hemoglobin

Fasting plasma glucose was determined using an enzymatic colorimetric assay (LABTEST GOD-ANA, Brasil). HbA1c was measured using HPLC (CLAE)

### Autoantibodies

Serum levels of GAD65A and IA-2A were determined by radioimmunoassay (RSR limited, UK; CV < 5.3%). The normal values of 700 healthy controls (considering 3 SD) were <1.0 IU/mL and <0.8 IU/mL for GAD65A and IA2A, respectively. Znt8A levels were measured by ELISA (Kronus, USA CV < 7%). *SLC30A8* polymorphisms at the codon for the 325^th^ amino acid result in the expression of three ZnT8 protein variants: arginine (R)325, tryptophan (W)325 and rarely glutamine (Q)325. ZnT8 autoantibodies are directed mainly at the C terminal domain of ZnT8 (residues 268–369). This assay detects and quantifies autoantibodies specific to R325, W325 or to residue 325 non-specific variants.

### *SLC30A8* genotyping

Genomic DNA was isolated from fresh peripheral blood cells by a conventional salting-out method^31^. The rs2466293 and rs16889462 variants were genotyped by the Vera Code Golden Gate (Illumina) methodology.

### Statistical analyses

The analyses were performed using SPSS^®^ 18.0, considering a p value lower than 0.05 as statistically significant.

Genotypic frequencies were calculated by allele counting, and the Hardy-Weinberg (H-W) equilibrium for each polymorphism was tested using a Chi-square test. Autoantibody measurements were compared among genotypes using Kruskal-Wallis or Mann-Whitney tests. The associations between the SNPs and diabetes mellitus risk were tested under three different genetic models: Genotypic Model (AA versus Aa versus aa), Dominant Model (AA versus Aa + aa) and Recessive Model (AA + Aa versus aa).

Chi-square or Fisher’s Exact tests were employed to compare the differences in the distribution of categorical variables (gender, ethnicity and genotype frequencies) between cases and controls and to associate autoantibody positivity and genotypes.

Moreover, the association between the polymorphisms and the risk of diabetes mellitus or anti-Zn positivity was estimated by odds ratios (ORs) and their 95% confidence intervals (95% CIs) using binary logistic regression analysis, which was adjusted for age, duration of disease, gender and ethnicity. Carriers of the wild-type genotype were used as a reference in this analysis.

The critical p value was corrected by Bonferroni adjustments and indicated as *Pc* for multiple tests. Non-parametric correlations (Spearman) were performed.
